# Reassessing the public health value of regional cancer registries in the era of Japan’s National Cancer Registry: a learning health system perspective

**DOI:** 10.3389/fonc.2026.1907140

**Published:** 2026-09-16

**Authors:** Hiroto Narimatsu, Kaname Watanabe, Choy-Lye Chei, Sho Nakamura

**Affiliations:** 1 Department of Genetic Medicine, Kanagawa Cancer Center, Yokohama, Japan; 2 Cancer Prevention and Control Division, Kanagawa Cancer Center Research Institute, Yokohama, Japan; 3 Graduate School of Health Innovation, Kanagawa University of Human Services, Kawasaki, Japan; 4 Center for Innovation Policy, Kanagawa University of Human Services, Kawasaki, Japan

**Keywords:** cancer screening, data matching system, effectiveness evaluation, Kanagawa prefecture, public health data infrastructure, quality control

## Abstract

Cancer registries are integral to public health infrastructure and support not only the monitoring of cancer incidence and mortality but also cancer control planning, quality assurance of screening programs, evaluation of healthcare delivery, prognostic assessment, and development of research infrastructure. Japan’s National Cancer Registry (NCR) was launched in 2016, and many long-standing regional cancer registries were subsequently integrated into the national system; however, the Kanagawa Cancer Registry (KCR) continues to operate independently. This Perspective examines how a regional cancer registry can retain public health value in the era of the NCR by serving as infrastructure for a Learning Health System (LHS) that supports cancer screening quality control, regional policy feedback, and continuous improvement. The KCR contributes to screening quality control, projection of cancer care demand, and identification of region-specific challenges that cannot be fully addressed by the NCR alone. In practice, an LHS cycle linking data use, evaluation, feedback, and improvement is gradually being established through the KCR. Thus, regional cancer registries in Japan should be reevaluated not merely as repositories of statistics, but as public health infrastructures that bridge regional policy and clinical practice.

## 1 Introduction

Cancer registries provide the basic statistics needed for understanding and, ultimately, controlling cancer. However, what is crucial for cancer control is not the collection of data itself, but the ability to use those data for simple statistics as well as policy formulation, research, and evaluation of regional healthcare systems. At the regional level, it is necessary to address issues specific to municipalities and regional medical service areas that are difficult to identify within a one-size-fits-all national system and to establish tailored strategies based on the specific characteristics of each municipality and medical region.

The National Cancer Registry (NCR) was implemented in Japan in 2016, marking the beginning of nationwide cancer data collection under a unified national framework. This registry represents a major institutional advance in Japanese cancer surveillance by providing a comprehensive, standardized nationwide statistical infrastructure. At the same time, many prefectural-level regional cancer registries that had played a pioneering role in regional cancer statistics ceased independent operations and were integrated into the NCR as data resources. Kanagawa Prefecture is the only prefecture that continues to operate an independent regional cancer registry. Maintaining a regional cancer registry does more than complement NCR data. In Kanagawa, the registry contains additional information, including TNM classification (tumor-node-metastasis staging information), which the NCR does not include, and functions as a regionally adaptable data foundation for a Learning Health System (LHS). An LHS is a framework that links regional data with research evidence and then feeds those findings back into practice to continuously improve quality, safety, and efficiency (1). The NCR is managed under a uniform national framework; therefore, the authority of regional stakeholders to modify operational procedures, data-use pathways, and feedback processes is inherently limited. In contrast, a regional cancer registry can adapt these processes to region-specific needs, allowing for more timely feedback, iterative refinement, and practical implementation; thus, it can function as infrastructure for an LHS.

The NCR was designed primarily as a population-based system for incidence and mortality surveillance rather than as a registry intended to capture the full range of detailed clinical information required for all forms of quality assessment. In this respect, the division of roles between population-based and clinically detailed data infrastructures is consistent with the design of comparable systems in other countries. Therefore, the present argument is not that the NCR is deficient because it does not serve all functions simultaneously, but that even within such a nationally centralized framework, a regionally embedded registry may retain distinct public health value when local linkage, context-sensitive implementation, and rapid feedback to practice and policy are required. Internationally, national cancer registry infrastructures are often accompanied by public-facing tools for information delivery, research access, and visualization. In the United States, for example, the CDC’s National Program of Cancer Registries (2), the SEER Program (3), and State Cancer Profiles (4) serve these functions. Japan’s NCR is likewise linked to official information resources through the National Cancer Center and the Cancer Information Service (4).

LHSs have gained increasing attention as a model for using routinely generated clinical and public health data to drive continuous improvement. At the national level, Singapore’s National Electronic Health Record (NEHR) (5) and Sweden’s National Quality Registries (6) provide examples of LHS implementation. At the municipal and regional levels, the Colorado Health Observation Regional Data Service, which covers seven counties in the Denver metropolitan area, is an example of regional public health LHS implementation (7). In addition, the Iowa Cancer Registry supported the development of a rural cancer network by generating hospital-specific and catchment-area reports and providing analytic and implementation support for quality improvement and accreditation activities in resource-constrained rural hospitals (8, 9). These examples suggest that public health data infrastructures can serve not only as surveillance systems, but also as operational platforms for coordination, feedback, and improvement. This perspective is directly relevant to regional cancer registries in Japan, whose potential extends beyond statistical data collection to serve as foundational infrastructures for an LHS. Together, these international examples support a broader principle relevant to the present Perspective: the public health value of a data infrastructure does not depend solely on national scale or data centralization, but also on its capacity to support locally actionable linkage, feedback, and iterative improvement.

At the program level, international experience also shows that effective cancer screening requires organized quality assurance supported by individual-level information systems and links to population-based registries. The CanScreen5 project has further emphasized the importance of routinely collecting and standardizing screening performance indicators to support monitoring, evaluation, and policy refinement across settings (10). In Japan, the development of cancer surveillance and evidence-based screening policy has likewise been described as a coordinated effort to standardize population-based registries, disseminate precise cancer information, and prevent the adoption of screening methods without scientifically established evidence of a favorable balance between benefits and harms (11). Although focused on cervical intraepithelial neoplasia and HPV, a recent narrative review similarly emphasized the integration of screening, follow-up, and equitable public health policy, supporting the broader relevance of registry-linked quality assurance and continuous improvement (12).

With the establishment of the NCR as a standardized foundation for nationwide cancer statistics collection in Japan, the distinctive public health value of regional cancer registries warrants renewed evaluation, particularly with respect to their capacity for context-sensitive implementation, feedback, and continuous improvement. This article explores the role that regional cancer registries, particularly the Kanagawa Cancer Registry (KCR), have played since the establishment of the national system from an LHS perspective and evaluates their public health value.

## 2 History and characteristics of the Kanagawa cancer registry

Kanagawa Prefecture is located south of Tokyo and has a population of approximately 9.2 million (14). Historically, population-based cancer registration in Japan began in Miyagi Prefecture in 1951 and expanded gradually across the country, reaching all 47 prefectures by 2013 (15). During this period, Osaka and Aichi established major regional cancer registries in 1962 (16, 17), and Kanagawa established its registry in 1970, making it one of the earliest such initiatives in Japan. After the enactment of the Act on Promoting Cancer Registries and the implementation of the NCR in 2016, the former prefectural registries were incorporated into a nationally standardized framework. Against this background, the KCR remains exceptional as an independently maintained regional registry.

The KCR was launched in 1970 as a joint project between the Kanagawa Medical Association and Kanagawa Prefectural Center for Adult Diseases (the predecessor of the present Kanagawa Cancer Center). Subsequently, in 1977, Kanagawa Prefecture assumed responsibility for its operations, which have remained independent since then. Following the implementation of the NCR, the KCR has remained the only independently maintained regional cancer registry in operation in Japan. According to the annual report of the KCR (13), the number of new cancer cases in Kanagawa Prefecture in 2022 was 72,801, accounting for approximately 6.8% of the national total.

The KCR has three distinctive features: additional data elements not routinely captured in the NCR, such as TNM classification; long-term, accumulated population-based data; and the flexible use of follow-up and prognosis-related information (**Table 1**). Furthermore, through sustained operations, practical working relationships centered on data use have been established among local governments, regional healthcare providers, and research communities. Therefore, the registry is not merely a data asset but a regionally embedded operational infrastructure supported by the experience and expertise accumulated through long-term management and use. In addition, the KCR has established an information security management system compliant with ISO/IEC 27001, which supports the secure management and operation of this regionally maintained data infrastructure. Consequently, the KCR can be evaluated as a core infrastructure for an LHS, supporting the identification of region-specific challenges, data linkage and analysis, feedback to frontline practice, and iterative operational improvement. Although some clinically detailed information is also captured through the Hospital-Based Cancer Registry (HBCR) system at designated cancer care hospitals, the KCR provides a complementary population-based and operational platform that can link data use more directly with regional public health practice.

**Table 1:** The national cancer registry and the Kanagawa cancer registry.

Item	National cancer registry (NCR)	Kanagawa cancer registry (KCR)
Governance	Nationally governed under the Cancer Registry Act and coordinated by the National Cancer Center	Regionally maintained in Kanagawa as an independent registry
Implementation	Established in 2016 as Japan’s national population-based cancer registry	Established in 1970 and continues to operate independently
Historical role	Unified the former prefectural population-based registries into a standardized national framework	Preserved the long-established regional registry infrastructure
Primary purpose	Nationwide cancer incidence surveillance and official cancer statistics	Regionally embedded public health applications, data linkage, feedback, and learning health system functions
Data design	Standardized national population-based data structure	Can support region-specific follow-up, local linkage, and flexible public health applications
Operational logic	National standardization and comparability	Regional adaptability and actionable local feedback

## 3 Current public health applications of the Kanagawa cancer registry

In the Kanagawa Prefecture Cancer Control Promotion Plan, the KCR has been integrated into several specific policy areas (18). First, the prefecture states that it will promote quality control of cancer screening in municipalities within the prefecture based on the “Cancer Screening Quality Control System built using regional cancer registry data,” indicating that the regional cancer registry serves as a data foundation for screening quality assurance. Second, the prefecture explicitly aims to use regional cancer registry data to conduct “simulations for projecting demand for cancer care” and to apply the findings to its cancer control measures. Third, for rare and intractable cancers, the use of cancer registries is recommended as a foundation for assessing information provision, such as case counts. Thus, the KCR serves as an information infrastructure supporting the prefecture’s cancer control measures in areas such as screening quality control, forecasting demand for cancer care, and identifying rare and intractable cancers.

The value of the registry is also reflected in the secondary use of its data. Ongoing applications from regional cancer centers, hospitals, universities, and other research institutions have been accepted (13). Furthermore, information on patient prognosis is frequently collected by regional medical institutions that report patient data to the KCR to complement hospital-based cancer registries, track long-term prognoses, and support the review of clinical practices.

It is important to distinguish the role of the KCR from that of the HBCR system. The HBCR, led by the National Cancer Center and implemented at designated cancer care hospitals, captures clinically detailed information such as cancer stage and treatment-related data and provides infrastructure for evaluating hospital-based cancer care. By contrast, the KCR is population-based and regionally embedded, allowing linkage across municipalities, hospitals, and other local stakeholders. Its distinctive contribution in the present context is therefore not merely the accumulation of detailed variables, but its capacity to support region-wide follow-up, screening quality control, and locally actionable feedback beyond the boundaries of individual hospitals.

A related distinction should be drawn between the KCR and the National Cancer Center’s Quality Indicator (QI) project. The national QI project uses linked hospital-based cancer registry and Diagnosis Procedure Combination (DPC) data to assess adherence to standard cancer care and other process indicators at the institutional level, and participating hospitals receive benchmarked feedback to support quality improvement. In contrast, the KCR-based activities described here address a different layer of quality improvement: they support population-based and municipally linked screening quality control, cross-sector operational feedback, and iterative refinement of regional public health practice. Thus, the KCR complements rather than duplicates the national QI effort.

Notably, the applications of KCR extend beyond academic secondary analyses to support administrative project evaluation and improvement in regional healthcare. Specific examples include assessing cancer screening performance by linking municipal cancer screening data, assessing changes in overall regional treatment outcomes and medical costs before and after the introduction of innovative treatments, and evaluating cancer diagnostics and treatment delays associated with the COVID-19 pandemic (13). Moreover, as stated in the Kanagawa Prefecture Cancer Control Promotion Plan, these uses extend beyond descriptive statistics to support policy decisions and program improvements. Consequently, the KCR can be viewed as an operational component of an LHS that supports local government decision-making and research.

## 4 Establishment of an infrastructure for LHS-oriented use: the cancer screening quality control and cancer screening effectiveness evaluation projects

Among the practical applications of the KCR, the Cancer Screening Quality Control Project in Yokohama City and the Cancer Screening Effectiveness Evaluation Project in Yokosuka City provide representative examples of its role as infrastructure for regionally embedded LHS. The former focuses on quality control of cancer screening in Yokohama City, while the latter evaluates the effectiveness of the ABC screening program in Yokosuka City. The ABC screening program is a risk-stratified approach to gastric cancer screening based on serum anti-*Helicobacter pylori* antibodies and pepsinogen testing. Both projects have been implemented in practice. Quality control of cancer screening refers to the systematic evaluation and improvement of screening programs through the linkage of screening records with cancer registry data. Such linkage is essential because valid estimation of key screening performance measures, including sensitivity and specificity, requires ascertainment of subsequent cancer occurrence among both individuals with positive screening results and those initially classified as screening-negative. In this context, cancer registry data enable the identification of false-negative cases and the use of these findings to improve screening programs. As shown in **Figure 1**, the Kanagawa model links municipal screening records, local name matching, ascertainment of subsequent cancer occurrences, estimation of screening performance, and feedback to local stakeholders within a continuous LHS cycle.

**Figure 1:**
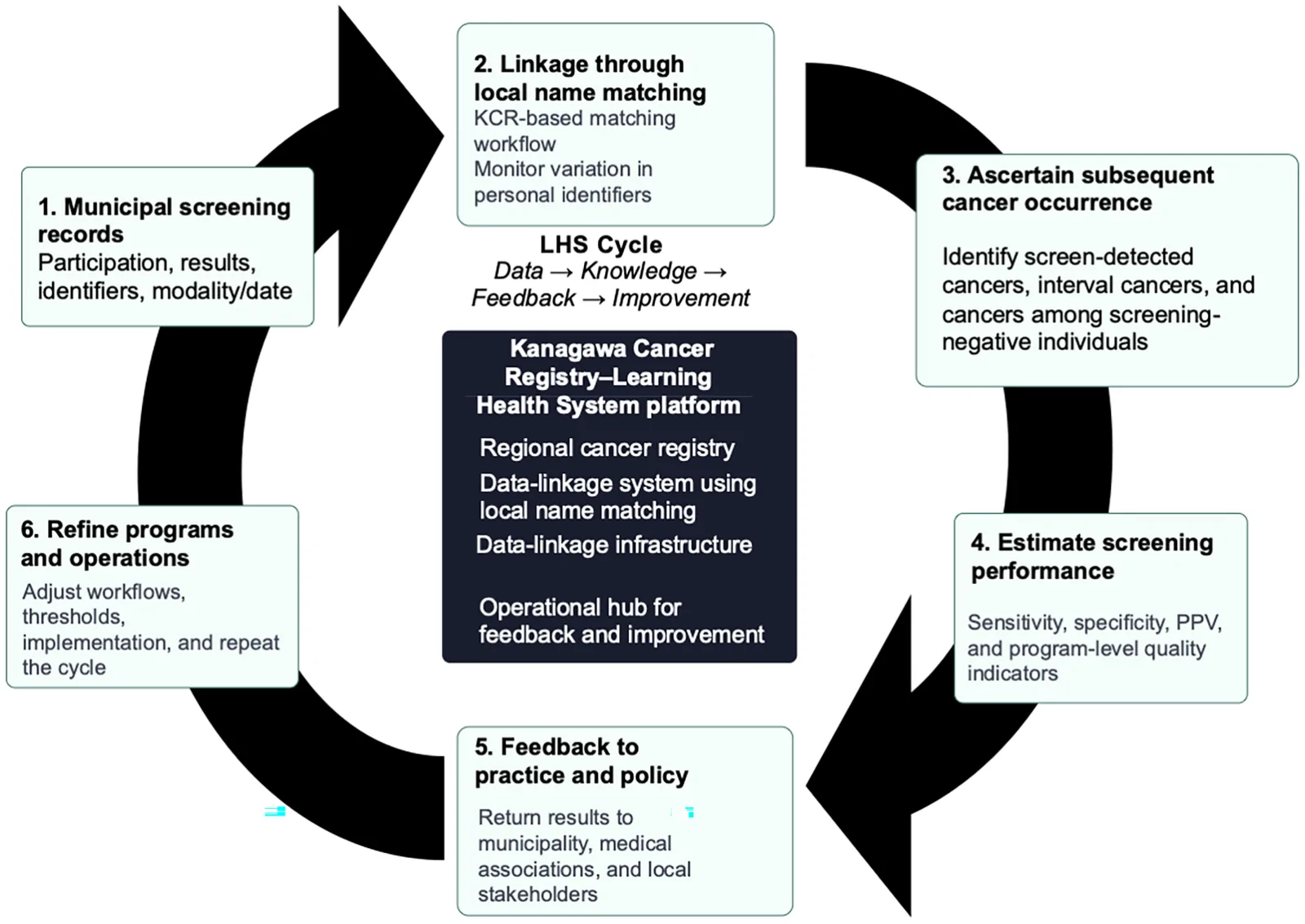
Learning health system cycle for cancer screening quality control. Municipal screening records were linked to the cancer registry data using local name matching to ascertain subsequent cancer occurrences, estimate screening performance, provide feedback to local stakeholders, and support iterative program improvement. Local name matching refers to a locally developed record-linkage process using personal identifiers. LHS, Learning Health System; PPV, positive predictive value.

The system supporting these operations is a locally developed data-linkage system using name matching, established through collaboration among the Yokohama Medical Association, Yokohama City, and Kanagawa Cancer Center. Using this system, a workflow comprising extraction from the master database, name-matching, and return of results has been established within the local environment. The development and operation of this system, which is tailored to regional data quality, data entry practices, and screening implementation systems, depends on a locally maintained data infrastructure. Because the NCR is administered under a nationally standardized, government-led framework, regional stakeholders have limited authority to modify operational procedures, data-use pathways, and feedback workflows to meet local needs. By contrast, a regional cancer registry allows these processes to be adapted to region-specific needs, thereby enabling more timely feedback and iterative improvement as part of an LHS-oriented infrastructure. These operational constraints limit the extent to which the NCR can replace the regional system for these functions. The NCR name-matching function is built into a web-based system, which does not allow a locally developed name-matching system to be used as an intermediary. In Japan, a universal personal identifier suitable for this type of record linkage is unavailable, and accurate linkage requires high-performance matching because of language-specific complexities in personal names. Moreover, some municipal datasets contain personal identifiers that cannot be adequately handled by a matching system approved under the NCR framework. In such situations, maintaining acceptable linkage quality would require extensive manual review of a large number of records, or would otherwise risk reducing matching accuracy. Consequently, maintaining the current operations solely within the NCR framework may be challenging in terms of time, cost, and accuracy. By contrast, local control within regional cancer registries enables system development, implementation, and practical use. Regional cancer screening programs vary widely, and local authorities can maintain and manage their own databases; therefore, the system can be developed and adapted to regional program requirements. Furthermore, a continuous improvement cycle can be established by incorporating feedback from medical associations into the operational processes. This feedback mechanism illustrates an LHS in practice. The Kanagawa model may serve as a useful reference not only within Japan, but also for other health systems seeking to combine centralized cancer surveillance with locally adaptable functions for record linkage, rapid feedback, and iterative quality improvement.

## 5 Discussion

This LHS model of the KCR, in which data are held locally and used in accordance with regional needs, faces challenges such as standardization, interoperability, auditing, allocation of responsibilities, and security risk management. However, these are not reasons for rejecting the decentralized regional model; rather, they are challenges that require appropriate management. The KCR has addressed these challenges through accumulated knowledge and close collaboration with medical institutions. Therefore, the assumption that a centralized system, such as the NCR, must serve as the sole model of cancer registry infrastructure should be reconsidered.

Maintaining regional databases can provide distinct public health value and the KCR can be regarded as a policy-relevant demonstration model. Regional registries can complement, and in some settings, provide a practical alternative for specific functions of the centralized model under unified national management, particularly where operational feasibility in real-world local settings, responsiveness, and regional adaptability are required. The KCR provides an example of the potential value of a regionally maintained data infrastructure for an LHS, even in the era of the NCR. Cancer InFocus provides a useful example of how centrally available data can be translated into regionally actionable tools for planning and quality improvement (19). While this platform is not equivalent to a regional cancer registry, it supports the broader point that national-scale data systems and regional implementation infrastructures can coexist productively. From this perspective, the NCR and the KCR should be viewed as complementary rather than competing systems: the former provides standardized nationwide surveillance, whereas the latter enables local linkage, contextual interpretation, and rapid feedback for region-specific public health action.

In conclusion, regional cancer registries should be reevaluated not simply as supplementary statistical resources, but as operational public health infrastructures that enable context-sensitive implementation, feedback, and continuous improvement. The experience of the KCR demonstrates that a regionally maintained registry can function as infrastructure for an LHS by linking screening records, outcome ascertainment, data analysis, and feedback to local stakeholders within a practical cycle of improvement. Although issues such as standardization, interoperability, governance, and security require careful management, these challenges do not diminish the value of regional systems; rather, they define the conditions under which regional and national infrastructures should complement one another. The KCR therefore represents a policy-relevant demonstration model suggesting that, even within centralized national registration, regionally embedded cancer registry infrastructures can play an important role in translating data into actionable improvements in cancer control. More broadly, regional registry infrastructures may retain substantial public health value alongside centralized national systems when local linkage, cross-sector coordination, and rapid feedback are necessary for effective action.

## Data Availability

The original contributions presented in the study are included in the article/supplementary material. Further inquiries can be directed to the corresponding author.
